# Racial Differences in the Impact of Subjective Life Expectancy on Advance Care Planning

**DOI:** 10.1093/geroni/igaa057.3325

**Published:** 2020-12-16

**Authors:** Yifan Lou, Deborah Carr

**Affiliations:** 1 Columbia University, New York, New York, United States; 2 Boston University, Boston, Massachusetts, United States

## Abstract

The need for advance care planning (ACP) is heightened during the COVID-19 pandemic, especially for older Blacks and Latinx persons who are at a disproportionate risk of death from both infectious and chronic disease. A potentially important yet underexplored explanation for well-documented racial disparities in ACP is subjective life expectancy (SLE), which may impel or impede ACP. Using Health and Retirement Study data (n=7484), we examined the extent to which perceived chances of living another 10 years (100, 51-99, 50, 1-49, or 0 percent) predict three aspects of ACP (living will (LW), durable power of attorney for health care designations (DPAHC), and discussions). We use logistic regression models to predict the odds of each ACP behavior, adjusted for sociodemographic, health, and depressive symptoms. We found modest evidence that SLE predicts ACP behaviors. Persons who are 100% certain they will be alive in ten years are less likely (OR = .68 and .71, respectively) whereas those with pessimistic survival prospects are more likely (OR = 1.23 and 1.15, respectively) to have a LW and a DPAHC, relative to those with modest perceived survival. However, upon closer inspection, these patterns hold only for those whose LW specify aggressive measures versus no LW. We found no race differences for formal aspects of planning (LW, DPAHC) although we did detect differences for informal discussions. Blacks with pessimistic survival expectations are more likely to have discussions, whereas Latinos are less likely relative to whites. We discuss implications for policies and practices to increase ACP rates.

